# Diet Quality of Pregnant American Indian Women in the Northern Plains

**DOI:** 10.5888/pcd16.180536

**Published:** 2019-04-25

**Authors:** Erin P. Ferranti, Terryl J. Hartman, Amy J. Elliott, Diane C. Mitchell, Jyoti Angal, Dana Nickleach, Moriah Bellissimo, Rosalind Breslow

**Affiliations:** 1 Nell Hodgson Woodruff School of Nursing, Emory University, Atlanta, Georgia; 2 Department of Epidemiology, Rollins School of Public Health, Winship Cancer Institute, Emory University, Atlanta, Georgia; 3 Avera Research Institute for Pediatric and Community Research, Sioux Falls, South Dakota; 4 Diet Assessment Center, Department of Nutritional Sciences, Penn State University, University Park, Pennsylvania; 5 Winship Cancer Institute, Emory University, Atlanta, Georgia; 6 Department of Medicine, Emory University, Atlanta, Georgia; 7 National Institute on Alcohol Abuse and Alcoholism, National Institutes of Health, Bethesda, Maryland

## Abstract

**Introduction:**

We examined diet quality and intake of pregnancy-specific micronutrients among pregnant American Indian women in the Northern Plains.

**Methods:**

We conducted an analysis of nutrition data from the Prenatal Alcohol and SIDS and Stillbirth (PASS) Network Safe Passage Study and the PASS Diet Screener study (N = 170). Diet intake, including dietary supplementation, was assessed by using three 24-hour recalls conducted on randomly selected, nonconsecutive days. Diet intake data were averaged across the participant’s recalls and scored for 2 dietary indices: the Healthy Eating Index 2010 (HEI-2010) and the Alternate Healthy Eating Index for Pregnancy (AHEI-P). We also assessed nutrient adequacy with Dietary Reference Intakes for pregnancy.

**Results:**

On average, participants were aged 26.9 (standard deviation [SD], 5.5) years with a pre-pregnancy body mass index of 29.8 (SD, 7.5) kg/m^2^. Mean AHEI-P and HEI-2010 scores (52.0 [SD, 9.0] and 49.2 [SD, 11.1], respectively) indicated inadequate adherence to dietary recommendations. Micronutrient intake for vitamins D and K, choline, calcium, and potassium were lower than recommended, and sodium intake was higher than recommended.

**Conclusion:**

Our findings that pregnant American Indian women are not adhering to dietary recommendations is consistent with studies in other US populations. Identifying opportunities to partner with American Indian communities is necessary to ensure effective and sustainable interventions to promote access to and consumption of foods and beverages that support the adherence to recommended dietary guidelines during pregnancy.

**Summary**

**What is already known on this topic?**

Suboptimal nutrition during pregnancy is a well-established risk factor for adverse maternal and fetal outcomes. Diet quality among pregnant women varies by demographic and psychosocial characteristics.

**What is added by this report?**

Similar to other populations in the United States, pregnant American Indian study participants are not adhering to recommendations for diet patterns and micronutrients recommended for pregnancy.

**What are the implications for public health practice?**

Assessment of dietary quality and micronutrient intake coupled with understanding the unique historical and contextual factors that contribute to eating behavior among pregnant American Indian women are needed to develop and test tailored interventions to ensure adequate diet quality and micronutrient supplementation during pregnancy.

## Introduction

Suboptimal nutrition during pregnancy is a well-established risk factor for adverse maternal and fetal outcomes (1). Adequate nutrition in pregnancy includes both overall diet quality and individual nutrient intake (2). Diet quality among pregnant women varies by demographic and psychosocial characteristics (3). Women with poor diet quality are more likely to have higher pre-pregnancy body mass index (BMI) and lower socioeconomic status and to be multiparous (4). Dietary patterns and micronutrient intakes also vary by race and ethnicity (5). American Indian women of reproductive age are at particularly high risk for poor health outcomes including hypertension, diabetes, obesity, and frequent mental distress (6). Despite these documented health disparities, little focus has been devoted to examining diet quality and nutrient intake during pregnancy in American Indian women.

In a study conducted more than 10 years ago that examined diet quality among low-income women participating in the Special Supplemental Nutrition Program for Women, Infants, and Children (WIC), both American Indian and white women reported suboptimal diet quality, and American Indian women reported significantly poorer quality as defined by the Diet Quality Index score for pregnancy (7). The poorer quality was characterized by unfavorable scores for intake of dietary cholesterol, total fat, and saturated fat. No recent study has reexamined diet quality in pregnant American Indian women. Therefore, the purpose of this study was to examine overall diet quality and intake of pregnancy-specific micronutrients during pregnancy among American Indian women in the Northern Plains.

## Methods

The Prenatal Alcohol and SIDS and Stillbirth (PASS) Network conducted the Safe Passage Study, a large, prospective study with enrollment from 2007 to 2015. The study was designed to examine the association between prenatal alcohol exposure and outcomes of stillbirth and sudden infant death syndrome. Pregnant women were recruited into the PASS study at 2 sites — the US Northern Plains and Cape Town, South Africa. A detailed description of the design, methods, recruitment, and follow-up of the PASS study is available (8). The PASS study was approved by multiple institutional review boards and tribal boards and was reviewed by an independent advisory and safety monitoring board. Data presented here are from women from the Northern Plains American Indian site who participated in an ancillary study to validate the PASS Northern Plains Diet Screener (9). This ancillary study was approved by the Sanford Health Institutional Review Board in Sioux Falls, South Dakota. The tribal communities represented here reviewed and approved the manuscript.

### Participants

Women presenting for a prenatal visit from 20 to 24 weeks gestation between June 2011 and May 2013 were asked to participate in this study. A total of 203 potentially eligible women were invited. Sixteen women declined, and 187 were enrolled; 170 had useable dietary data for this analysis. The ancillary study was described, and informed consent was obtained. Demographic and clinical data about the participants, including maternal age, gestational age, pre-pregnancy BMI (calculated from self-reported weight [kg]/height [m^2^]), smoking, and educational status, were available from the parent study.

### Dietary assessment and scoring

Dietary intake, including dietary supplementation, was assessed by three 24-hour recalls, collected on randomly selected, nonconsecutive days (2 on weekdays and 1 on a weekend day) by unannounced telephone calls from trained nutrition personnel. Participants were provided a booklet that depicted food amounts to assist them in visual cues in reporting quantities, and the diet interviewers asked whether the 24-hour recall represented a day that was typical, lower, or higher than normal. The dietary recalls were analyzed using the computer-directed software Nutrition Data System for Research (University of Minnesota). The Nutrition Coordinating Center Food and Nutrient Database at the University of Minnesota includes approximately 18,000 foods and contains food items representative of American Indian diets. The data were averaged across the participant’s recalls; scored for 2 dietary indices, the Healthy Eating Index 2010 (HEI-2010) and the Alternate Healthy Eating Index for Pregnancy (AHEI-P) (3); and examined for nutrient adequacy using the Dietary Reference Intakes (DRIs) for pregnancy (10).

HEI-2010 is a 12-component dietary index that quantifies the concordance with the 2010 Dietary Guidelines for Americans (11). The 12 components are total fruit, whole fruit, total vegetables, greens and beans, whole grains, dairy, total protein foods, seafood and plant proteins, fatty acids, refined grains, sodium, and empty calories (eg, added sugars). Each component is awarded a minimum score of 0 and a maximum score of 5 to 20 points, so the total score for the HEI-2010 ranges from 0 to 100. The higher the score, the more adherent the diet is to the guidelines.

The AHEI-P was modified from the Alternate Healthy Eating Index (AHEI) (12) to incorporate pregnancy-specific components (3). It is a 9-component scale in which each component is scored from 0 to 10 points, for a maximum score of 90. Higher scores indicate better adherence with dietary recommendations for pregnancy. Modifications from the original AHEI include the exclusion of alcohol, because it is not recommended during pregnancy, and the exclusion of nuts and soy as protein components, because many women refrain from eating nuts during pregnancy due to allergy concerns; the soy protein component is instead included as tofu or soybeans in the vegetable component. Finally, dietary intakes of 3 nutrients, folate, iron and calcium, are added as individual components because of their importance during pregnancy. The full 9 components of the AHEI-P are vegetables, fruit, ratio of white to red meat, fiber, *trans* fatty acids, ratio of polyunsaturated to saturated fatty acids, folate, calcium, and iron.

The DRIs are the nutrient-based reference values that guide the definition of nutrient adequacy and dietary guidelines (10). Depending on the evidence, an estimated average requirement, a recommended dietary allowance (RDA), or an adequate intake is established for each nutrient. Many nutrients also have a tolerable upper intake level. Parameters for each nutrient are specific to sex, age, and life stage. The estimated average requirement estimates the needs of half the healthy individuals in a particular sex, age, or life-stage group, whereas the RDA is the average daily intake sufficient to meet the nutrient requirements of 97% to 98% of healthy individuals in a particular sex, age, or life-stage group. When an RDA cannot be determined, the adequate intake is indicated, and this represents the recommended nutrient intake level based on an approximation or estimation assumed to be adequate. Finally, the upper intake level represents the highest average daily nutrient intake level likely to pose no risk of adverse health effects to most healthy individuals in the sex, age, or life-stage group. For most nutrients, there are established estimated average requirement, RDA or adequate intake and upper intake level recommendations for pregnancy at different maternal ages (14–18 y, 19–30 y, and 31–50 y) (10).

### Data analysis

After calculating the index-specific dietary variables, we examined univariate statistics and distributions of all variables and the mean values for each dietary component by the HEI-2010 and AHEI-P indices. We then examined the percentage of participants who met the minimum and maximum scores for each dietary component. We also calculated the group mean intake of nutrients and compared these to the DRI, RDA, or adequate intake for pregnant women aged 19 to 30 years and 31 to 50 years. Dietary supplemental intake was also examined and added to the nutrient intake to compute total average daily intake and determine the percentage of participants meeting the RDA or adequate intake for pregnancy. Finally, we conducted bivariate analyses to examine demographic and clinical associations with mean diet quality scores. All analyses were conducted using SAS version 9.3 (SAS Institute, Inc).

## Results

Approximately 65% of participants had at least a high school education and 58% reported being married. Mean pre-pregnancy BMI was 29.8 (standard deviation [SD], 7.5), indicating that most women were overweight entering pregnancy. A high proportion (55%) of women reported current smoking during their pregnancy.

Mean score for the HEI-2010 was 49.2 (SD, 11.1) (Table 1) and for the AHEI-P was 52.0 (SD, 9.0) (Table 2). Areas of greatest deficiency were in total vegetable intake and green vegetable intake. Other concerns were poor fatty acid ratio, poor white meat to red meat ratio and high intakes of empty calories and sodium. Intake of both iron and folate was low. Most participants met the criteria for maximum score of total protein intake (HEI-2010).

**Table 1:** Mean Daily Dietary Intake of Pregnant Northern Plains American Indian Women (N = 170), Measured Against Healthy Eating Index 2010**^a^** Criteria, United States, 2011–2013

Component	Mean (SD) Intake, by Component Criteria	Maximum Score	Criteria for Minimum Score of 0	Criteria for Maximum Score	Participant Scores, Mean Points (SD)^b^	Participants Who Met Criteria for Minimum Score, n (%)	Participants Who Met Criteria for Maximum Score, n (%)
Total fruit	0.45 (0.51)	5	No fruit	≥0.8 cup equivalent/1,000 kcal	2.3 (1.8)	24 (14.1)	28 (16.5)
Whole fruit	0.17 (0.22)	5	No whole fruit	≥0.4 cup equivalent/1,000 kcal	1.8 (1.8)	56 (32.9)	22 (12.9)
Total vegetables	0.68 (0.31)	5	No vegetables	≥1.1 cup equivalent/1,000 kcal	3.0 (1.2)	0	16 (9.4)
Greens and beans	0.05 (0.08)	5	No dark-green vegetables, beans or peas	≥0.2 cup equivalent/1,000 kcal	1.1 (1.7)	92 (54.1)	12 (7.1)
Whole grains	0.97 (0.92)	10	No whole grains	≥1.5 oz equivalent/1,000 kcal	5.3 (3.5)	16 (9.4)	36 (21.2)
Dairy	0.86 (0.49)	10	No dairy	≥1.3 cup equivalent/1,000 kcal	6.1 (2.7)	2 (1.2)	24 (14.1)
Total protein foods	2.54 (0.85)	5	No protein foods	≥2.5 oz equivalent/1,000 kcal	4.4 (0.9)	0	95 (55.9)
Seafood and plant proteins	0.37 (0.74)	5	No seafood or plant proteins	≥0.8 oz equivalent/1,000 kcal	1.4 (1.9)	89 (52.4)	23 (13.5)
Fatty acids	1.71 (0.41)	10	(PUFAs + MUFAs)/SFAs ≤1.2	(PUFAs + MUFAs)/SFAs ≥2.5	2.6 (2.1)	13 (7.6)	6 (3.5)
Refined grains	2.62 (0.88)	10	≥4.3 oz equivalent/1,000 kcal	≤1.8 oz equivalent/1,000 kcal	6.7 (3.8)	28 (16.5)	67 (39.4)
Sodium	1.80 (0.31)	10	≥2.0 g/1,000 kcal	≤1.1 g/1,000 kcal	2.85 (2.5)	32 (18.8)	2 (1.2)
Empty calories	31.82 (7.23)	20	≥50% of energy	≤19% of energy	11.7 (4.4)	3 (1.8)	5 (2.9)

Abbreviation: MUFAs, monounsaturated fatty acids; PUFAs, polyunsaturated fatty acids; SD, standard deviation; SFAs, saturated fatty acids.

a Each component of the Healthy Eating Index 2010 is awarded a minimum score of 0 and a maximum score of 5 to 20 points; the total score ranges from 0 to 100.

b The total score of participants was 49.2 (SD, 11.1) points.

**Table 2:** Mean Daily Dietary Intake of Pregnant Northern Plains American Indian Women (N = 170), Measured Against Alternate Healthy Eating Index for Pregnancy Criteria, United States, 2011–2013

Component	Mean Intake (SD)	Criteria for Minimum Score	Criteria for Maximum Score	Participant Scores, Mean (SD) Points	Participants Who Met Criteria for Minimum Score, n (%)	Participants Who Met Criteria for Maximum Score, n (%)
Vegetables, servings/d	1.8 (1.1)	0	≥5	3.6 (2.2)	1 (0.6)	4 (2.4)
Fruit, servings/d	1.8 (1.8)	0	≥4	4.0 (3.2)	24 (14.1)	17 (10.0)
Ratio of white to red meat	0.73 (2.2)	0	≥4	1.5 (2.3)	53 (31.2)	4 (2.4)
Fiber, g/d	17.6 (6.8)	0	≥25	6.8 (2.3)	0	23 (13.5)
*trans* Fat, % of energy	1.4 (0.6)	≥4	≤0.5	7.6 (1.6)	0	1 (0.6)
Polyunsaturated to saturated fat ratio	0.69 (0.25)	≤0.1	≥1.0	6.4 (2.3)	0	26 (15.3)
Calcium, mg/d	978.4 (332.2)	0	≥1,200	7.7 (1.9)	1 (0.6)	31 (18.2)
Folate, µg/d	512.2 (178.7)	0	≥600	7.9 (1.8)	0	42 (24.7)
Iron, mg/d	18.5 (5.8)	0	≥27	6.7 (1.7)	0	15 (15.0)
Total score	—	0	90	52.0 (9.0)	—	—

Abbreviation: —, not applicable.

Intake of micronutrients from food and drink, excluding supplementation, were also suboptimal (Table 3). Most participants (90%) reported use of at least one dietary supplement and when supplemental intakes were added to dietary intake, vitamins D, E, K, folate, pantothenic acid, choline, calcium, magnesium, and potassium were all less than recommended intake for more than half of the participants (Table 3). Intake of sodium was high. Only 8.2% women consumed less than the upper intake level of 2,300 mg per day, and only 1.2% were compliant with the RDA of less than or equal to 1,500 mg per day.

**Table 3:** Mean Daily Dietary Intake of Pregnant Northern Plains American Indian Women (N = 170), Measured Against Micronutrient Dietary Reference Intakes for Pregnancy, United States, 2011–2013

Nutrient	Dietary Intake, Mean (SD)	Combined Dietary and Supplement Intake, Mean (SD)	Recommended Dietary Allowance or Adequate Intake for Pregnancy	Participants Who Met RDA or Adequate Intake Criteria (%)
Vitamin A, µg/d	735.9 (529.6)	2,751.3 (919.1)	770	96.0
Vitamin C, mg/d	104.2 (81.0)	222.9 (137.9)	85	93.5
Vitamin D, µg/d	5.5 (3.5)	17.394 (32.5)	15	48.2
Vitamin E, mg/d	11.1 (6.9)	55.8 (25.5)	15	84.1
Vitamin K, µg/d	74.3 (41.5)	75.0 (41.6)	90	25.3
Thiamin, mg/d	1.6 (0.9)	2.0 (0.74)	1.4	98.2
Riboflavin, mg/d	2.3 (1.0)	3.9 (1.3)	1.4	98.8
Niacin, mg/d	24.9 (8.9)	42.0 (11.0)	18	97.6
Vitamin B_6_, mg/d	2.1 (0.84)	4.6 (2.3)	1.9	96.5
Folate, µg/d	512.2 (242.5)	1,208.7 (360.7)	600	94.7
Vitamin B_12,_ µg/d	5.8 (3.8)	21.6 (85.1)	2.6	98.8
Pantothenic acid, mg/d	5.3 (2.5)	6.4 (4.3)	6	35.9
Choline, mg/d	341.0 (152.2)	343.2 (152.1)	450	16.5
Calcium, mg/d	977.8 (469.3)	1,127.9 (561.0)	1,000	60.0
Copper, µg/d	1,270.0 (663.0)	1,423.6 (887.6)	1,000	70.0
Iron, mg/d	18.5 (8.2)	48.4 (25.2)	27	89.4
Magnesium, mg/d	261.8 (93.3)	272.6 (112.9)	350 (if aged 19–30 y) 360 (if aged 31–50 y)	18.2
Manganese, mg/d	3.2 (1.4)	3.3 (1.4)	2.0	81.2
Phosphorous, mg/d	1,319.1 (490.4)	1,324.9 (491.0)	700	94.1
Selenium, µg/d	122.3 (45.3)	123.2 (45.4)	60	97.6
Zinc, mg/d	12.9 (5.4)	33.5 (10.2)	11	96.5
Potassium, mg/d	2,642.5 (977.9)	2,644.0 (978.7)	4,700	4.1
Sodium, mg/d	4,049.0 (1,592.5)	NA	1,500	8.2

Abbreviations: NA, not applicable; RDA, recommended dietary allowance; SD, standard deviation.

When we examined bivariate associations with mean diet quality, we found no significant association with pre-pregnancy BMI or education level. Only older age demonstrated a weak positive correlation with both AHEI-P mean scores (*r* = .16, *P* = .05) and HEI-2010 mean scores (*r* = .16, *P* = .04).

## Discussion

We examined overall diet quality and intake of micronutrients during pregnancy among American Indian women in the Northern Plains. Our findings that pregnant American Indian women have suboptimal diet patterns are consistent with findings in many other US populations, both pregnant and not pregnant. Diet patterns in other American Indian populations (13) have been characterized by high intakes of processed meats, fried foods, and sodium, and low intakes of fruits, vegetables, and whole grains. Findings from the largest longitudinal study of dietary intake conducted in American Indian populations, the Strong Heart Dietary Study, indicated that suboptimal diet quality is common among American Indians; however, it is comparable to the inadequate diet quality patterns of the general US population (13). This finding was also confirmed in the American Indian populations participating in the Special Diabetes Program for American Indians Diabetes Prevention Demonstration Project (14). Although American Indian diets in general are not optimal, few differences were found in comparison to the overall US population.

There are indicators that overall diet quality may be improving in subsets of the US population. HEI-2010 scores are trending upward, and the most recent 2011–2012 National Health and Nutrition Examination Study (NHANES) sample averaged 59.0 points (15). Examined by race/ethnicity, HEI-2010 scores and the 2007–2010 NHANES differed; the other/mixed race group had the highest diet quality score (mean, 60.7; 95% confidence interval [CI], 58.5–63.0), followed by the non-Hispanic white group (mean, 56.2; 95% CI, 54.4–58.1); it was lowest among the non-Hispanic black group (mean = 51.0; 95% CI, 49.8–52.2) (16). The authors did not report diet quality scores for American Indians.

In a more recent study where the HEI-2010 was used to assess diet quality of a large cohort of pregnant women who completed a semiquantitative food frequency questionnaire (n = 8,259), the mean score was 63 (SD, 13) points; non-Hispanic black and Hispanic women scored lower than non-Hispanic white women: 54 (SD, 11) points and 61 (SD, 12) points, respectively, compared with 65 (SD, 12) points (17). When comparing the HEI-2010 scores of these cohorts to our sample with an HEI-2010 mean score of 49.2, it is clear that there is a need to address overall diet quality in American Indian women.

Our study raises particular concern for the health of American Indian women and their babies, especially those with lower-than-recommended intakes of micronutrients. Our findings, combined with similar findings in a study published over a decade ago in a comparable population of American Indian pregnant women (7), demonstrate that little progress has been made in ensuring adequate diet quality in this population. Findings by Watts and colleagues determined that diets among both low-income white women and American Indian women were deficient in intakes of folate, iron, grains, vegetables, and whole fruit (7) — areas where we also found deficiencies. Also notable in our study sample were that more than half of the women reported smoking during pregnancy, which not only is associated with adverse maternal and fetal outcomes but also increases requirements of micronutrients such as vitamin C (18).

There is significant variability in diet quality, as measured by AHEI-P scores, across studies of pregnant women. Differences in diet assessment methods may in part contribute to differences between samples. A recent nationally representative study of pregnant US women participating in NHANES concluded that mean AHEI-P scores were 41.9 of a modified 80-point scale, or a score of 52.4% (19). Our finding of 52 on a 90-point scale, or a score of 57.7%, suggests that the population of American Indian pregnant women in our study has an overall higher diet quality, though still suboptimal, compared with the general US population. One consideration is that NHANES participants typically contribute one or 2 days of dietary data rather than 3, as in our study, which could allow for more influence of days of unusual dietary intake (eg, poor diet quality) in NHANES. In contrast, compared with another study of racially diverse pregnant women from the Project Viva study, where mean AHEI-P scores calculated from a food frequency questionnaire were 61.0 (SD, 10.0), our population of American Indian women scored nearly 10 points lower on the AHEI-P. Taken together, it appears that pregnant American Indian women have suboptimal diet quality that is not markedly different from other populations of pregnant US women.

In our study, some key pregnancy-specific micronutrient levels, including vitamin D, folate, and iron, were not achieved with dietary intake alone. However, after adding in supplementation, recommendations for these micronutrients were met, highlighting the importance of supplementation during pregnancy to meet recommended intakes. No other recent studies have reported dietary micronutrient intake in pregnant American Indian women. In nonpregnant American Indian women, intake of micronutrients including calcium, magnesium, potassium, vitamin A, vitamin D, and vitamin E have been reported as suboptimal (20). Among other racial/ethnic minority pregnant populations, such as African American women, dietary micronutrient intake is inadequate, particularly for folate, vitamin D, iron, and choline (21).

Although our findings support previous work indicating that there is little to no substantial difference in diet quality patterns or micronutrient intake in American Indian populations compared with other US subpopulations, focus should be on improving diet quality in American Indians because of the significantly higher rates of type 2 diabetes and cardiovascular disease, both of which occur in American Indian populations at twice the rate of the US general population, and cardiometabolic disparities in morbidity and mortality rates are increasing (22,23). Furthermore, American Indian women have a disproportionate burden of adverse maternal and infant health outcomes, including higher rates of preterm birth and gestational diabetes (24). Improving diet quality is an important strategy to address these health disparities in American Indian women.

Our study was limited to describing overall diet quality in a subpopulation of pregnant American Indian women and was not able to explore factors that may influence eating behavior. However, other researchers have summarized strategies to address healthy dietary patterns in American Indian populations, highlighting local and federal initiatives to promote access to locally grown, affordable foods and beverages (25). These initiatives include funding opportunities that are using population-specific guidelines to address sociocultural factors in expanding healthy food access. Historically, American Indian populations, particularly those who live in rural areas, had limited access to fresh food (26). A recent report found a food insecurity rate of 28% in the Northern Plains region; food insecurity continues to be a challenge among many American Indian communities (27). Where food assistance programs are available, it appears that food packages in the Food Distribution Program on Indian Reservations met more of the HEI-2010 recommendations than other federal food assistance and nutrition programs (28). However, the food packages scored poorly in the areas where we also found deficiencies: total fruit, total vegetables, and greens and beans (28). Beyond simply improving food access and quality, sustainable and effective improvements in American Indian dietary patterns should focus on solutions that increase tribal control of food production and access (29). Furthermore, to address factors that influence eating behavior of pregnant American Indian women, additional research in this population is needed.

Dietary intake was assessed via self-report, which is subject to underreporting and recall bias. To minimize error and bias, we used three 24-hour detailed dietary recalls, conducted by unannounced telephone calls from trained nutrition personnel, 2 on weekdays and 1 on a weekend day. In community-based cohort studies, 24-hour diet recalls have been shown to be less biased than other dietary assessment methods, are culturally neutral, and provide greater detail about foods consumed (30). Dietary supplement use was also included and was essential in determining adequacy of micronutrient intake during pregnancy. This was a descriptive analysis of a subset of 170 women participating in a larger study that aimed to examine the association between prenatal alcohol exposure and outcomes of stillbirth and sudden infant death syndrome (8). Because the parent study did not collect detailed dietary intake data suitable for diet pattern characterization, we were limited to a descriptive analysis of the diet pattern and self-reported supplement intake in this subset. We did not have access to data to further understand potential reasons for the suboptimal dietary intake and low self-reported intake of prenatal supplemental vitamins. Therefore, further study is warranted to investigate barriers to high quality dietary patterns and supplemental vitamin intake among pregnant American Indian women.

Similar to other populations in the US, pregnant American Indian study participants are not adhering to recommendations for diet patterns and micronutrients recommended for pregnancy. Assessment of dietary quality and micronutrient intake coupled with understanding the unique historical and contextual factors that contribute to eating behavior among pregnant American Indian women are needed to develop and test tailored interventions to ensure adequate diet quality and micronutrient supplementation during pregnancy.

## References

[1] Gresham E , Bisquera A , Byles JE , Hure AJ . Effects of dietary interventions on pregnancy outcomes: a systematic review and meta-analysis. Matern Child Nutr 2016;12(1):5–23. 10.1111/mcn.12142 25048387 PMC6860081

[2] Murphy MM , Stettler N , Smith KM , Reiss R . Associations of consumption of fruits and vegetables during pregnancy with infant birth weight or small for gestational age births: a systematic review of the literature. Int J Womens Health 2014;6:899–912. 10.2147/IJWH.S67130 25349482 PMC4208630

[3] Rifas-Shiman SL , Rich-Edwards JW , Kleinman KP , Oken E , Gillman MW . Dietary quality during pregnancy varies by maternal characteristics in Project Viva: a US cohort. J Am Diet Assoc 2009;109(6):1004–11. 10.1016/j.jada.2009.03.001 19465182 PMC4098830

[4] Bodnar LM , Siega-Riz AM . A Diet Quality Index for Pregnancy detects variation in diet and differences by sociodemographic factors. Public Health Nutr 2002;5(6):801–9. 10.1079/PHN2002348 12570888

[5] Brunst KJ , Wright RO , Digioia K , Enlow MB , Fernandez H , Wright RJ , Racial/ethnic and sociodemographic factors associated with micronutrient intakes and inadequacies among pregnant women in an urban US population. Public Health Nutr 2014;17(9):1960–70. 24476840 10.1017/S1368980013003224 PMC4071127

[6] Amparo P , Farr SL , Dietz PM . Chronic disease risk factors among American Indian/Alaska Native women of reproductive age. Prev Chronic Dis 2011;8(6):A118. 22005611 PMC3221560

[7] Watts V , Rockett H , Baer H , Leppert J , Colditz G . Assessing diet quality in a population of low-income pregnant women: a comparison between Native Americans and whites. Matern Child Health J 2007;11(2):127–36. 10.1007/s10995-006-0155-2 17191147

[8] Dukes KA , Burd L , Elliott AJ , Fifer WP , Folkerth RD , Hankins GD , ; PASS Research Network. The Safe Passage Study: design, methods, recruitment, and follow-up approach. Paediatr Perinat Epidemiol 2014;28(5):455–65. 10.1111/ppe.12136 25131605 PMC4286367

[9] Hartman TJ , Elliott AJ , Angal J , Block T , Ferranti EP , Mitchell DC , Relative validation of a short questionnaire to assess the dietary habits of pregnant American Indian women. Food Sci Nutr 2016;5(3):625–32. 28572950 10.1002/fsn3.440 PMC5448387

[10] Institute of Medicine. Dietary reference intakes: applications in dietary assessment. Washington (DC): The National Academies Press; 2000. 25057725

[11] Guenther PM , Kirkpatrick SI , Reedy J , Krebs-Smith SM , Buckman DW , Dodd KW , The Healthy Eating Index-2010 is a valid and reliable measure of diet quality according to the 2010 Dietary Guidelines for Americans. J Nutr 2014;144(3):399–407. 10.3945/jn.113.183079 24453128 PMC3927552

[12] McCullough ML , Feskanich D , Stampfer MJ , Giovannucci EL , Rimm EB , Hu FB , Diet quality and major chronic disease risk in men and women: moving toward improved dietary guidance. Am J Clin Nutr 2002;76(6):1261–71. 10.1093/ajcn/76.6.1261 12450892

[13] Conti K . Nutrition status of American Indian adults and impending needs in view of the Strong Heart Dietary Study. J Am Diet Assoc 2008;108(5):781–4. 10.1016/j.jada.2008.02.022 18442499

[14] Teufel-Shone NI , Jiang L , Beals J , Henderson WG , Zhang L , Acton KJ , Demographic characteristics and food choices of participants in the Special Diabetes Program for American Indians Diabetes Prevention Demonstration Project. Ethn Health 2015;20(4):327–40. 10.1080/13557858.2014.921890 24954106 PMC5108238

[15] Wilson MM , Reedy J , Krebs-Smith SM . American diet quality: where it is, where it is heading, and what it could be. J Acad Nutr Diet 2016;116(2):302–10 e301. 26612769 10.1016/j.jand.2015.09.020 PMC4733413

[16] Rehm CD , Monsivais P , Drewnowski A . Relation between diet cost and Healthy Eating Index 2010 scores among adults in the United States 2007–2010. Prev Med 2015;73:70–5. 10.1016/j.ypmed.2015.01.019 25625693 PMC4869858

[17] Bodnar LM , Simhan HN , Parker CB , Meier H , Mercer BM , Grobman WA , Racial or ethnic and socioeconomic inequalities in adherence to national dietary guidance in a large cohort of US pregnant women. J Acad Nutr Diet 2017;117(6):867–877.e3. 10.1016/j.jand.2017.01.016 28320597 PMC5446928

[18] Northrop-Clewes CA , Thurnham DI . Monitoring micronutrients in cigarette smokers. Clin Chim Acta 2007;377(1-2):14–38. 10.1016/j.cca.2006.08.028 17045981

[19] Gamba R , Leung CW , Guendelman S , Lahiff M , Laraia BA . Household food insecurity is not associated with overall diet quality among pregnant women in NHANES 1999–2008. Matern Child Health J 2016;20(11):2348–56. 10.1007/s10995-016-2058-1 27406151

[20] Ali R , Lee ET , Knehans AW , Zhang Y , Yeh J , Rhoades ER , Dietary intake among American Indians with metabolic syndrome — comparison to dietary recommendations: the Balance Study. Int J Health Nutr 2013;4(1):33–45. 26594109 PMC4651460

[21] Groth SW , Stewart PA , Ossip DJ , Block RC , Wixom N , Fernandez ID . Micronutrient intake is inadequate for a sample of pregnant African-American women. J Acad Nutr Diet 2017;117(4):589–98. 10.1016/j.jand.2016.11.011 28065633 PMC5367978

[22] Go AS , Mozaffarian D , Roger VL , Benjamin EJ , Berry JD , Blaha MJ , ; American Heart Association Statistics Committee and Stroke Statistics Subcommittee. Heart disease and stroke statistics — 2014 update: a report from the American Heart Association. Circulation 2014;129(3):e28–292. 10.1161/01.cir.0000441139.02102.80 24352519 PMC5408159

[23] Centers for Disease Control and Prevention. National diabetes statistics report, 2017: estimates of diabetes and its burden in the United States. Atlanta (GA): US Department of Health and Human Services; 2017.

[24] Anderson KG , Spicer P , Peercy MT . Obesity, diabetes, and birth outcomes among American Indians and Alaska Natives. Matern Child Health J 2016;20(12):2548–56. 10.1007/s10995-016-2080-3 27461020 PMC5124395

[25] Fleischhacker S . Emerging opportunities for registered dietitian nutritionists to help raise a healthier generation of Native American youth. J Acad Nutr Diet 2016;116(2):219–25. 10.1016/j.jand.2015.10.018 26680608 PMC4733391

[26] Kaufman P , Dicken C , Williams R . Measuring access to healthful, affordable food in American Indian and Alaska Native tribal areas. Washington (DC): US Department of Agriculture; 2014.

[27] Jernigan VBB , Huyser KR , Valdes J , Simonds VW . Food insecurity among American Indians and Alaska Natives: a national profile using the current population survey-food security supplement. J Hunger Environ Nutr 2017;12(1):1–10. 10.1080/19320248.2016.1227750 28491205 PMC5422031

[28] Byker Shanks C , Smith T , Ahmed S , Hunts H . Assessing foods offered in the Food Distribution Program on Indian Reservations (FDPIR) using the Healthy Eating Index 2010. Public Health Nutr 2016;19(7):1315–26. 10.1017/S1368980015002359 26298513 PMC5439495

[29] Echo Hawk Consulting. Feeding ourselves: food access, health disparities, and the pathways to healthy Native American communities 2015. http://nebula.wsimg.com/891e74d1afe847b92abe87b2a1df7c63?AccessKeyId=2EF8ECC329760AC5A98D&disposition=0&alloworigin=1. Accessed March 6, 2017.

[30] Subar AF , Freedman LS , Tooze JA , Kirkpatrick SI , Boushey C , Neuhouser ML , Addressing current criticism regarding the value of self-report dietary data. J Nutr 2015;145(12):2639–45. 10.3945/jn.115.219634 26468491 PMC4656907

